# Enantioselective supramolecular devices in the gas phase. Resorcin[4]arene as a model system

**DOI:** 10.3762/bjoc.8.62

**Published:** 2012-04-12

**Authors:** Caterina Fraschetti, Matthias C Letzel, Antonello Filippi, Maurizio Speranza, Jochen Mattay

**Affiliations:** 1 Sapienza University of Rome, Department of Chemistry and Technologies of Drug, Piazzale Aldo Moro, 5, 00185, Rome, Italy; 2 Bielefeld University, Department of Chemistry, Organic Chemistry I, P.O. Box 100131, 35501 Bielefeld, Germany

**Keywords:** diastereomeric complexes, gas phase enantioselectivity, kinetics, mass spectrometry, resorcin[4]arene receptor

## Abstract

This review describes the state-of-art in the field of the gas-phase reactivity of diastereomeric complexes formed between a chiral artificial receptor and a biologically active molecule. The presented experimental approach is a ligand-displacement reaction carried out in a nano ESI-FT-ICR instrument, supported by a thermodynamic MS-study and molecular-mechanics and molecular-dynamics (MM/MD) computational techniques. The noncovalent ion–molecule complexes are ideal for the study of chiral recognition in the absence of complicating solvent and counterion effects.

## Review

Enzymes are macromolecular assemblies that make up the machinery whose structures and dynamics enable and support life functions. They are invariably characterized by more-or-less flexible structures with asymmetric cavities of appropriate shape and size possessing suitable functionalities in specific positions. The large number of existing enzymes characterized by a specific function has provided chemists with both the stimulus and inspiration to design “synthetic enzymes” in order to provide exemplars suitable for improving the understanding of the amazing properties of natural biomolecules and for attempts to reproduce them for practical applications. Thus, noncovalent complexes between chiral receptors and biomolecules represent an important class of life's supramolecular systems in which the guest molecule (e.g., amino acids, neurotransmitters, drugs) is selectively captured into the host macromolecular structure (molecular recognition) and transformed catalytically at a specific “active site” (enzyme catalysis) [1–3]. Furthermore, the biorecognition may require the partial or complete desolvation of the guest molecule and of the polar groups of the active site by greatly enhancing its reactivity [4].

An important step towards the elucidation of enzyme mechanisms requires a comprehensive study of the structure, dynamics, and reactivity of simplified models under conditions, such as the gas phase, in which the noncovalent interactions in the guest–host complex are not perturbed by effects owing to the medium. As biological function and morphology are strongly correlated, knowledge of the supramolecular host–guest structures is expected to shed light on their biological functions. From the beginning of evolutionary processes right up to the present biodiversity, life relies on biological specificity, which arises from the fact that individual biomolecules “communicate” through noncovalent interactions. Resorcin[4]arenes are an important class of tunable macrocycles largely studied in the context of host–guest chemistry, as cavitands [5] and capsules [6]. The great ability of resorcin[4]arenes to trap several classes of compounds makes them very suitable for the subtle study of the chemicophysical properties of their host–guest systems, even in the gas phase. The most recent advances in this field include several gas-phase investigations: (i) The size and structure selectivity of tetraethyl and tetraphenyl resorcin[4]arenes in the recognition of mono, di-, and oligosaccharides by electrospray coupled with Fourier transform ion cyclotron resonance mass spectrometry (FT-ICR) [7]; (ii) the gaseous (and solution) selectivity towards several organic and inorganic anions [8], and tetramethylphosphonium cation [9]; and (iii) the complexation of saturated, nonsaturated, and aromatic dicarboxilic acids by a tetraammonium C1-resorcinarene, strongly dependent on the isomeric structure of the used guest [10].

Resorcin[4]arene molecules are characterized by three main contact regions [11]: (1) The *down*-region is the cavity of the receptor, which can be hydrophilic or lipophilic depending on the nature of the lateral chains; (2) The *external*-region is located in the proximity of lateral chains; and (3) The *up*-region is defined by the upper rim of the receptor crown. Furthermore, the nature of the pendants allows for subtle tuning of the polarity of both the *down*- and the *external*-regions, and the size of the *up*-region. The capability to discriminate biomolecules such as the zwitterionic forms of aromatic amino acids [12], basic amino acids [13–15], aliphatic and aromatic native amino acids [16–18], and amines and peptides [19], by acting as an artificial receptor [20–30], is thanks to this structural versatility.

The kinetic measurement of ligand-displacement reactions [31–37] is one of the different mass-spectrometric approaches used to promote an efficient chiral recognition [38], as already mentioned in the last review published in this field [31]. In the present review the attention will be focussed on the most recent results obtained by our group with this particular kinetic method.

### Methodology

The proton-bound [***M***∙H∙G]^+^ aggregates (***M***: chiral hosting resorcin[4]arene; G: guest biomolecule) were generated by electrospray ionization (ESI) of ***M***/G methanolic mixtures (where the ***M*** to G ratio lies in the range of 0.1–1), and then transferred into the resonance cell of a FT-ICR mass spectrometer by using an accumulation hexapole and two gradients: (1) The electrostatic gradient was kept by a system of potentials and lenses, and (2) the pressure gradient was maintained by a differential pumping system along the ion trajectory. The proton-bound [***M***∙H∙G]^+^ complex of interest was isolated by broad-band ejection of the other ions and then quenched by collisions with an inert gas (e.g., methane, argon), which pulsed into the cell through a magnetic valve. After the thermalization, the complex was allowed to stay in the cell for a variable reaction delay and then made to collide with a chiral or achiral reagent B introduced into the cell at a fixed pressure (10^−10^ to 10^−8^ mbar, Equation 1).

[1]
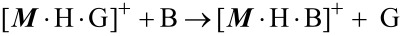


**Figure 1:**
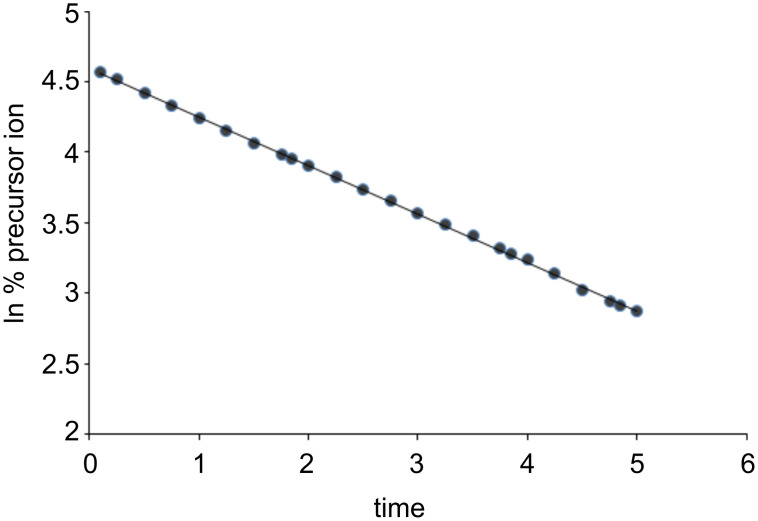
Examples of monoexponential decay: The slope of the line directly provides the reaction pseudo-first-order rate constant.

The extraction of the ligand-exchange rate constant is based on the decay of the isolated precursor ion [***M***∙H∙G]^+^ as a function of time *t*. If *I* is the intensity of the precursor [***M***∙H∙G]^+^ at the delay time *t* and *I*^0^ is the sum of the signals of [***M***∙H∙G]^+^ and [***M***∙H∙B]^+^, a monoexponential ln(*I*/*I*^0^) versus *t* plot is often obtained, whose slope provides the pseudo-first-order rate constant *k*_exp_ for the reaction in Equation 1. The monoexponential decay of an isolated system indicates that either just one reacting species exists or that more than one structure exists but that they react with similar rate constants (different species with a rate-constant ratio of less than 10 are kinetically indistinguishable, Figure 1).

The bimolecular rate constant (*k*_bi_) in monoexponential kinetics was calculated by solving Equation 2:

[3]
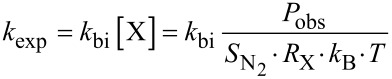


[2]
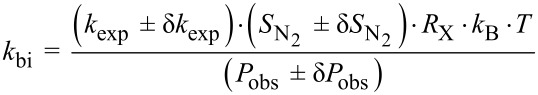


*P*_obs_: Observed pressure corrected for the background pressure.


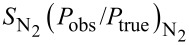
: Chemical sensitivity of vacuum gauge for N_2_.


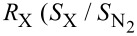
; reacting gas dependent): Calibration factor of the vacuum gauge.

*k*_B_ = Boltzmann constant

**Figure 2:**
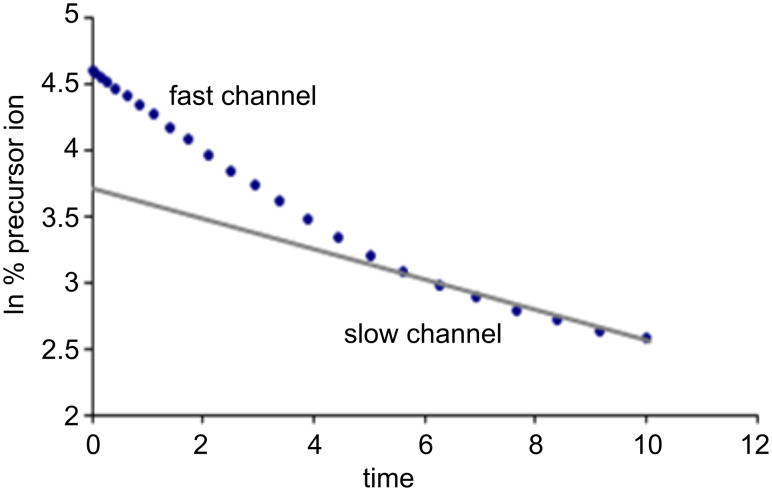
Example of biexponential decay.

Less frequently a biexponential decay was observed. This kinetic behavior indicates the presence of at least two different reacting structures: One of them decays faster (*k*_fast_) than the other (*k*_slow_: Figure 2).

In the latter case the following expression was used:

[4]
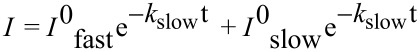


*I*^0^_fast_ = intensity of the fast reacting structure at *t* = 0

*I*^0^_slow_ = intensity of the slow reacting structure at *t* = 0

*I*^0^_slow_ is extractable from the intercept of the slow component with the *y*-axis, with its slope giving *k*_slow_. At this point Equation 4 also provides *k*_fast_, considering that *I*^0^_fast_ + *I*^0^_slow_ = 100. The bimolecular *k_(_*_bi)fast_ and *k*_(bi)slow_ are obtained from *k*_fast_ and *k*_slow_ by using Equation 2. Finally, the calculated *k*_bi_ were compared with the thermal capture rate with the neutral bath (*k*_cap_) in order to obtain the efficiency of the reaction as *k*_bi_/*k*_cap_ × 100. For this purpose the ion was treated as a point charge and the polar molecule as a two-dimensional rigid rotor by using the classical trajectory model (CT) of Su and Chesnavich [39–40].

When the host and the guest in the complex have the same absolute configuration, the rate constant of reaction described by Equation 1 is denoted as *k*_homo_; when instead they have opposite configuration, the rate constant is denoted as *k*_hetero_. The kinetic enantioselectivity of Equation 1 is obtained by comparing the second-order rate constants *k* for the same reaction involving the diastereomeric [***M***∙H∙G]^+^_homo_ and [***M***∙H∙G]^+^_hetero_ complexes, by means of the ρ factor (= *k*_homo_/*k*_hetero_). Furthermore, when the guest exchange of Equation 1 involves a chiral reactant B (either B_S_ or B_R_), another enantioselectivity factor ξ can be extracted from the kinetic results, based on the ratio of the rate constants of the same reaction involving B_R_ (*k*_R_) and B_S_ (*k*_S_), namely ξ = *k*_R_/*k*_S_. Obviously a ρ > 1 value indicates that the reactant B displaces the guest from the homochiral complex faster than from the heterochiral one. The opposite is true when ρ < 1, whereas a ρ = 1 indicates a lack of enantioselectivity. Analogously, a ξ > 1 value indicates that the displacement of the guest from a given complex is faster with B_R_ than with B_S_. Again, the opposite is true when ξ < 1. A ξ = 1 value corresponds to equal displacement rates irrespective of the configuration of B.

Chiral calixarenes, and their resorcinarene relatives, can be characterized by a variable conformational flexibility. They may exist in a highly symmetric bowl-shaped conformation, a so-called cone conformation, or in several other asymmetric conformations. In general, the chirality of resorcin[4]arenes can be due to (1) the presence of stereogenic centers in their side chains, or (2) the hindered spatial arrangement of achiral subunits forming a chiral macrocyclic scaffold.

### Chiral centers in the side chains

**Figure 3:**
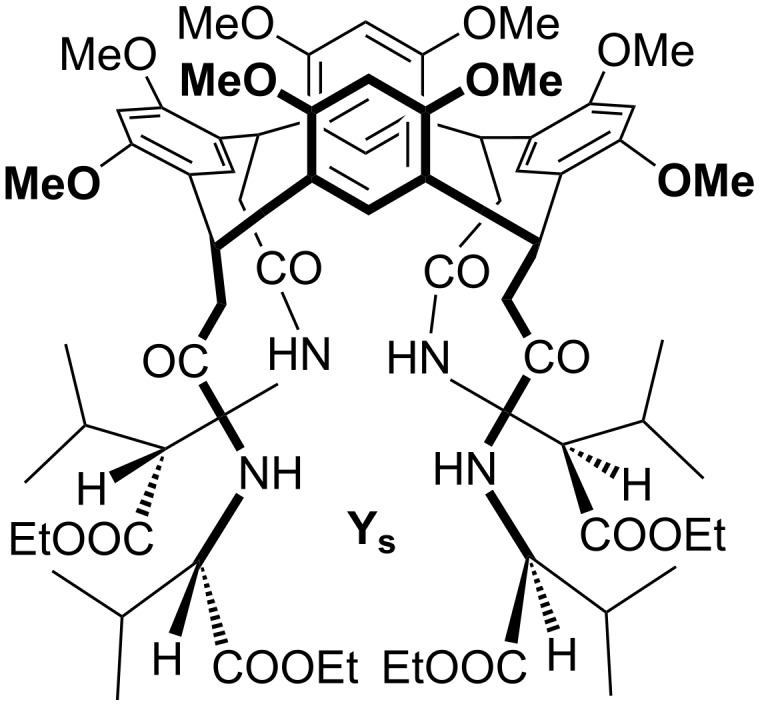
Amidoresorcin[4]arene **Y_S_**.

**Flexible peptidoresorcin[4]arenes as chiral selectors of dipeptides.** In 2002, the first investigations of chiral recognition by calixarenes in the gas phase were carried out in Rome by the group of Prof. Speranza by using ESI-FT-ICR-MS. They published several other studies [11,41–42] on the displacement of selected amino acids (G) from a type-1 chiral amidoresorcin[4]arene **Y_S_** (Figure 3) whose molecular asymmetry is due to the four axial pendants containing the chiral L-valine group. Two chiral effects were experimentally considered: The configuration of the amine B and that of the guest G. According to theoretical calculations, the chiral effects are insignificant if the guest molecule in the [**Y_S_**∙H∙G]^+^ complex is located outside the cavity of the host, while a bimodal kinetic is mirrored by the coexistence of a different guest position depending on the G configuration.

**Scheme 1:**
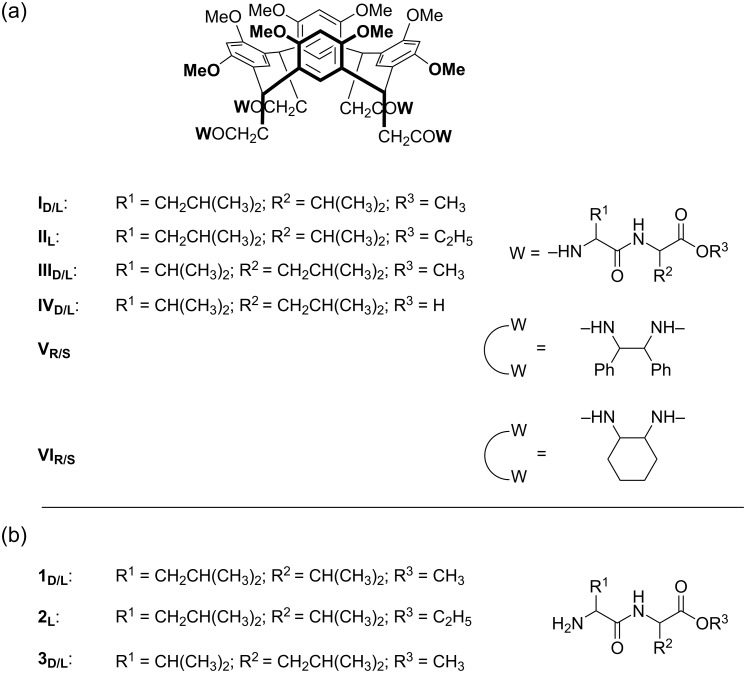
Studied (a) peptidoresorcin[4]arenes and (b) dipeptidic guests.

In 2009, the lengths and the complexity of the lateral chains were modified in order to investigate the effect of the nature, and the sequence of the *N*-linked amino acid residues in the *down*-region of the host. The resorcin[4]arene octamethyl ethers were functionalized with leucyl-valine and valyl-leucine (**I** and **III**; Scheme 1a) methyl esters, and the enantioselectivity toward the same dipeptide esters used in their synthesis, namely, leucyl-valine-OMe and valyl-leucine-OMe (**1** and **3**; Scheme 1b) was investigated [43].

A configurational preference was pointed out from previous NMR experiments [44]: In CDCl_3_ solution the neutral homochiral aggregate is significantly more stable than the heterochiral one, while NMR 1D ROESY results indicated that the dipeptidic guest is not located in the cavity of the host, but that the interaction that is mainly involved is hydrogen bonding occurring on the external surface of the resorcin[4]arene. These preliminary results motivated the gas-phase enantioselectivity study reported in [43], in which the guest displacement between proton-bound diastereomeric [***M***∙H∙G]^+^ (***M***: **I**–**IV**; G = **1**–**3**) complexes and (*R*)-(−)-2-butylamine (B) (Equation 5 and Equation 6) was monitored. In addition to the displacement enantioselectivity, the structural analogies between the resorcinarene and the dipeptides made other evaluations possible as, for instance, the effect of the –R in the CO_2_R groups of the host and the guest (–CH_3_ versus –C_2_H_5_), and the aminoacidic-sequence effect of the host pendants and the guest.

[5]
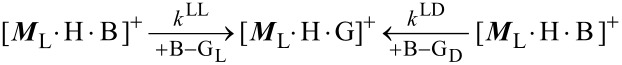


[6]
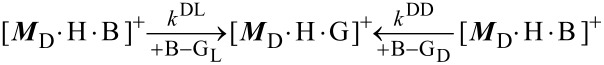


The [***M***∙H∙G]^+^ (***M***: **I**–**III**) complexes invariably reveal linear ln(*I*/*I*^0^) versus *t*. In contrast, biexponential kinetics are observed with [**IV**∙H∙G]^+^. As pointed out in the methodology paragraph, the latter kinetic behavior is ascribed to the occurrence of at least two stable isomeric [***M***∙H∙G]^+^ structures, one less reactive ([**IV**∙H∙G]^+^_slow_) and the other more reactive ([**IV**∙H∙G]^+^_fast_). The configuration of B does not appreciably influence the reactions kinetics of Equation 5 and Equation 6 [43], thus suggesting that the amine does not need to enter the cavity to interact with the chiral lower portion of the host before displacing the dipeptide G from the complex. This hypothesis agrees well with the external location of G in the [***M***∙H∙G]^+^ complex that was observed in independent solution experiments [44].

The heterochiral [***M***∙H∙G]^+^ (***M***: **I**–**III**) complexes react more efficiently than their homochiral analogues, with the exception of the “slow” population of the heterochiral [**IV**∙H∙**3**]^+^. Both the populations of the diastereomeric [**IV**∙H∙**1**]^+^ react with scarce or absent enantioselectivity.

Concerning the structural effect of the ester tail, the kinetics of the reactions in Equation 5 and Equation 6 are appreciably affected by the nature of the –CO_2_R function of the dipeptidic guest, while they are not subject to the influence of the specific ester functions of the host pendants [43], thus, providing further evidence that the guest is placed outside the host cavity in [***M***∙H∙G]^+^ (***M*** = **I**–**III)**. The negligible effect observed between [**IV**∙H∙G]^+^_fast_ and their [**III**∙H∙G]^+^ analogues (G = **1**, **3**) was correlated to a similar spatial arrangement of the guests with respect to their receptors, while the relatively slow population of [**IV**∙H∙G]^+^ (G = **1**, **3**) reacts with an efficiency that is definitely lower than [**III**∙H∙G]^+^. This diverging behavior suggests that the guest in [**IV**∙H∙G]^+^_slow_ is arranged in a completely different orientation, and this particular feature is due to the presence of the COOH tail in **IV** pendants, which acts as a protonated “hook” for dipeptides [43]. Indeed, the reaction enantioselectivity factors reflect these differences ([***M***∙H∙G]^+^ (***M*** = **I–III**): ρ < 1; [**IV**∙H∙G]^+^_slow_: ρ ≥ 1). Based on the NMR measurements [44], in which the homochiral [***M***∙H∙G]^+^ complexes appear to be more stable than the heterochiral ones, a ρ < 1 factor measured with [***M***∙H∙G]^+^ (***M*** = **I**–**III**) was rationalized from a thermodynamic point of view. The most enantioselective receptor towards guests **1** and **3** was **III**, while the lowest selectivity was observed with their [**I**∙H∙3]^+^ analogues. Irrespective of the nature of the host pendants (**III** or **I**), the complex of the guest **3** is more reactive than those of **1**, with the only exception of the heterochiral [**I**∙H∙G]^+^ (G = **1**, **3**). This enantioselectivity trend was ascribed to structural and steric factors, given the similar basicity of **1**–**3** [45–47]. The access of the amine B to the supramolecular assembly may be influenced by both steric and orientation factors, which can split the thermodynamic stability of the complexes and/or determine the dynamics of the displacement. In summary, the kinetic results [43] indicate that a dipeptidic guest is located outside the cavity of an analogous resorcin[4]arene, with the NH_2_ terminus coordinated by the amido group of a pendant and the estereal terminus H-bonded to the adjacent pendant. When the estereal tail is substituted by a –CO_2_H group, the hydrogen-bonding network is deeply modified. This gas phase arrangement, reproducing quite well the previous NMR experiments, is strongly influenced by the configuration of the partners in the charged aggregate.

**Figure 4:**
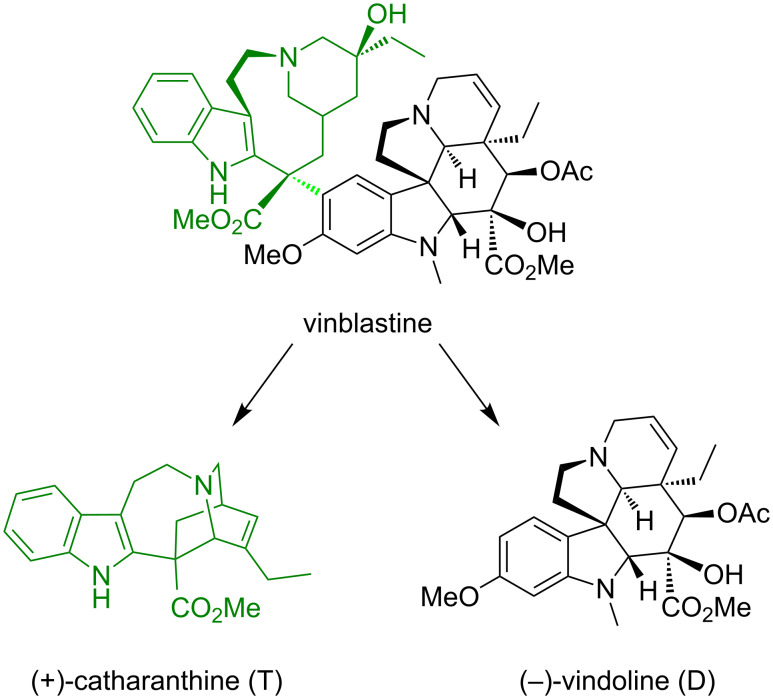
Catharanthine and vindoline, monomers constituting the anticancer vinblastine and the analogous vincristine.

**Flexible peptidoresorcin[4]arenes as chiral selectors of vinca alkaloids.** The interactions of the vinca alkaloids with the same resorcin[4]arenes were investigated in order to shed some light on the origin of the anticancer activity, by focusing on the drug/receptor interaction. Vinblastine and vincristine are “dimeric” molecules, comprising two subunits, i.e., rearranged (+)-catharanthine (T) and (−)-vindoline (D) (Figure 4) [48]. The gas-phase ligand-displacement approach was employed to investigate the intrinsic properties of these monomers on a molecular level [49]. First of all **I**, **III**, and **IV** were employed as artificial receptors characterized by more flexible lateral chains. The effect of the skeleton rigidity was evaluated by further investigation of the kinetic behavior of the rigid **V** and **VI** resorcin[4]arenes as chiral receptors.

The reaction with (*R*)-(−)-2-butylamine as the neutral gas B exhibited a significant enantioselectivity (Table 1).

**Table 1:** Exchange rate constants (*k* × 10^−11^ cm^3^ molecule^−1^ s^−1^).

Complex	ρ

[**I_D_**∙H∙T]^+^_homo_	1.70 ± 0.22
[**I_L_**∙H∙T]^+^_hetero_	

[**III_D_**∙H∙T]^+^_homo_	1.02 ± 0.14
[**III_L_**∙H∙T]^+^_hetero_	

[**IV_D_**∙H∙T]^+^_homo_	0.70 ± 0.14
[**IV_L_**∙H∙T]^+^_hetero_	

[V**_R_**∙H∙T]^+^_homo_	16.9 ± 2.8
[V**_S_**∙H∙T]^+^_hetero_	

[V**I_R_**∙H∙T]^+^_homo_	0.56 ± 0.07
[V**I_R_**∙H∙T]^+^_homo_	

In all cases the displacement reaction of [***M***∙H∙T]^+^ and [***M***∙H∙D]^+^ (***M*** = **I**–**III**) exhibited a monoexponential decay, while [**V_R/S_**∙H∙D]^+^ diastereoisomers react by following a bimodal kinetic. When the COOMe terminus was replaced by the COOH group ([**IIIV_D/L_**∙H∙T]^+^ → [**VI_D/L_**∙H∙T]^+^) an appreciable increase of enantioselectivity in the B-to-T displacement process was observed, while the inversion of the pendant sequence ([**I_D/L_**∙H∙T]^+^ → [**III_D/L_**∙H∙T]^+^) induced a significant reduction of the reaction enantioselectivity. The investigated [***M***∙H∙D]^+^ complexes basically showed enantioselective parallels to that of the corresponding [***M***∙H∙T]^+^ adduct.

**Figure 5:**
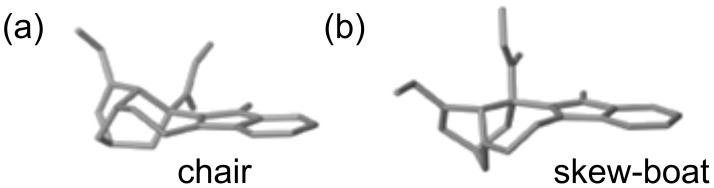
Stable conformers of catharanthine.

**Rigid resorcin[4]arenes as chiral selectors of vinca alkaloids.** Further attention was focused on the diastereomeric [**V_R/S_**∙H∙T]^+^ complexes whose large enantioselectivity (ρ = 16.9 ± 2.8) must be essentially promoted by substantial differences in the relevant reaction pathway [49]. Indeed, from the computational analysis performed on catharanthine (T), two families of conformers resulted as stable from the study performed in vacuum as well as in water: The skew-boat conformation of catharanthine is about 1 kcal mol^−1^ more stable than the chair conformation (Figure 5).

The skew-boat structure of both T and TH^+^ is persistent during all the MD simulations, while the chair→skew-boat interconversion very easily occurs in aqueous TH^+^. Furthermore, in all simulations the position of the CO_2_Me function oscillates between two orientations differing by 180°, except for TH^+^ in vacuum, while the ethyl group is invariably free to rotate in the three-dimensional space [49].

The computational results indicated that the chair conformation is better stabilized by the solvation and torsional energy terms than the skew-boat one is. In contrast, the electrostatic factor may induce a stronger stabilization of the skew-boat minimum, surpassing all the other effects. It can be concluded that in vacuum, electrostatic interactions prevail against the other energy terms, such that the T molecule, and the TH^+^ ion even more, are locked in the skew-boat. When the dielectric constant becomes high (water), torsional and solvation factors may become comparable to intramolecular electrostatic factors, with the consequence that conformational flexibility of the structure may increase.

**Figure 6:**
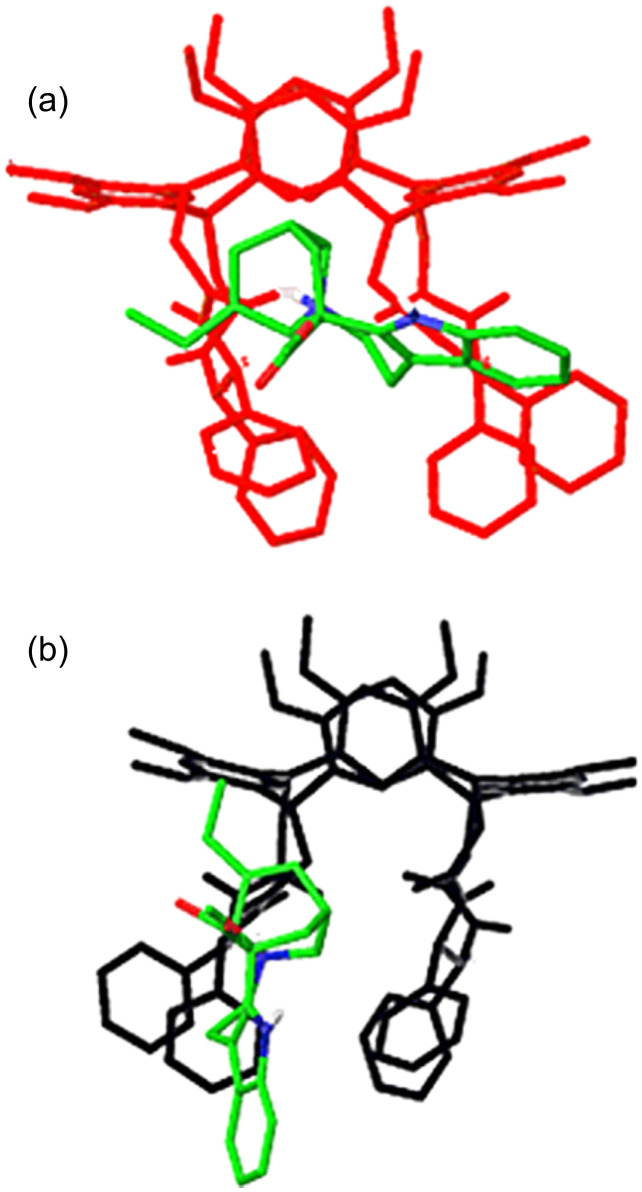
Global minima of (a) [**V_S_**∙H∙T]^+^ and (b) [**V_R_**∙H∙T]^+^ complexes.

According to the MM and MD calculations, both the [**V_S/R_**∙H∙T]^+^ enantiomers of the host tend to orientate two adjacent carbonyl oxygen atoms to the basic site of the guest. This arrangement requires that the structure of the host is strongly distorted from the uncomplexed form, by formation of a modified intramolecular hydrogen-bonding network at the lower rim [49]. The hosting regions of resorcin[4]arenes appear to be one the mirror image of the other, as does the hydrogen-bonding network involved in the interaction. Nevertheless, the marked differences, which finally justify the exceptional enantioselectivity measured, concern the orientation of the catharanthine in the lower rim of the cavity (Figure 6).

An aspect deserving more attention is the relative energies of the diastereomeric minima, which are very similar to each other, and thus this excludes an important contribution of thermodynamic control in the FT-ICR-MS experiments. The large enantioselectivity could be explained on the grounds of the pre-exponential term of Arrhenius, because depending on the orientation of catharanthine in the cavity there is more or less space for the approaching amine, which thus strongly influences the effective probability that the necessary proton transfer from T to B occurs.

**Figure 7:**
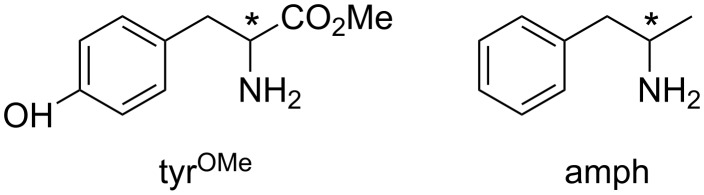
Guests studied in [47].

**Rigid resorcin[4]arenes as chiral selectors of amino acids and neurotransmitters.** Further insights into the molecular recognition of basket resorcin[4]arene **V** towards representative chiral molecules were gathered. For this purpose, the proton-bonded diastereomeric [**V**∙H∙G]^+^ complexes [G = tyrosine methyl ester (tyr^OMe^), and amphetamine (amph); Figure 7] were generated in the ESI source of an FT-ICR-MS to measure the kinetics of their ligand displacement towards the enantiomers of the neutral 2-aminobutane (B) (Table 2) [50].

**Table 2:** Exchange rate constants (*k* × 10^−10^ cm^3^ molecule^−1^ s^−1^).

[***M***∙H∙G]^+^	B = (*R*)-(–)-C_4_H_9_NH_2_	B = (*S*)-(+)-C_4_H_9_NH_2_
	ρ *= k*_homo_/*k*_hetero_	ρ *= k*_homo_/*k*_hetero_

[**V**∙H∙tyr^OMe^]^+^	0.93 ± 0.03	0.78 ± 0.04
[**V**∙H∙amph]^+^	1.26 ± 0.09	0.91 ± 0.06

Irrespective of the configuration of B the [**V**∙H∙tyr^OMe^]^+^_hetero_ complex reacts faster than its homochiral counterpart. In contrast, the selectivity of the amphetamine aggregate strictly depends on the configuration of the approaching amine, as it shows ρ > 1 factors in the reaction with B_R_ and ρ < 1 factors in that with B_S_.

To verify whether the measured enantioselectivity is determined by the relative stability of the starting diastereomeric complexes or by the transition states involved in the reaction path, the collision-induced dissociation spectra of the [**V**∙H∙tyr^OMe^]^+^ and [**V**∙H∙amph]^+^ complexes were recorded ([**V**∙H]^+^ is the unique CID fragment, arising from the loss of the guest molecule) [50]. The *R* [51] values obtained in the CID of the [**V**∙H∙tyr^OMe^]^+^ and [**V**∙H∙amph]^+^ complexes are definitely far from unity (0.32 and 0.48, respectively). These results indicate that both the homochiral [**V**∙H∙tyr^OMe^]^+^ and [**V·**H**·**amph]^+^ complexes are significantly more stable than their heterochiral analogues. Based on the CID results, it can be concluded that the displacement reaction of the [**V·**H**·**tyr^OMe^]^+^ complexes is mainly controlled by the relative stability of the starting diastereoisomers, while the ligand-exchange enantioselectivity of [**V·**H**·**amph]^+^ is determined by the effects of the chiral resorcin[4]arene scaffold upon the transition-state structures involved in the reaction, similarly to the complex of **V** with catharanthine.

**Figure 8:**
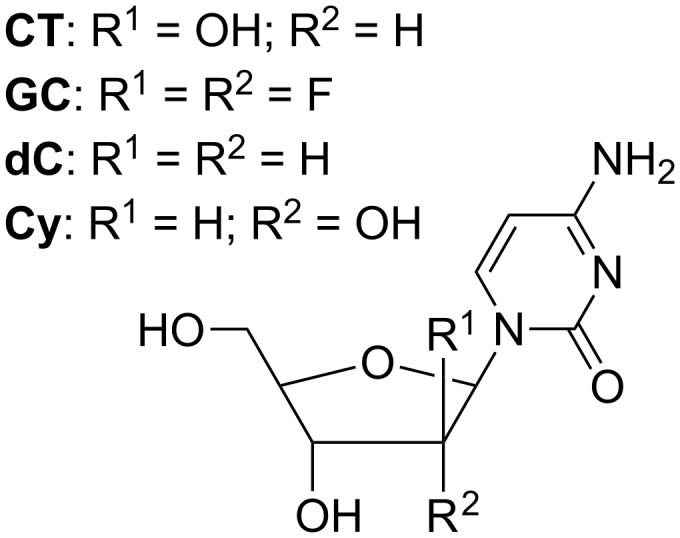
Selected nucleosides.

**Rigid resorcin[4]arenes as chiral selectors of nucleosides.** Nucleosides are the elementary units of the RNA and DNA biomacromolecules, and their physiological importance at many different levels [52–54] makes them potential candidates as anticancer drugs. The gas-phase study of the intimate interactions between the resorcin[4]arene **V** and several pyrimidine nucleosides can be an inspiration for both the design of new drug carriers, characterized by high solubility and selectivity, and a better understanding of the selective uptake of nucleosides by their respective membrane receptors [55]. The selected pyrimidine nucleosides are reported in Figure 8, i.e., 2’-deoxycytidine dC, cytidine, Cy, cytarabine CT, an epimer of cytidine, and gemcitabine GC, which is the *gem*-difluoro derivative of 2’-deoxycytidine (Figure 8).

Two reaction products were observed in the reaction with the enantiomers (2)-aminobutane: the addition [***M*·**H**·**G**·**B]^+^ and the ligand-exchange [***M*·**H**·**B]^+^ derivative. The experimental data was successfully fitted by using the integrated equation describing the kinetic Equation 7 [55].

[7]
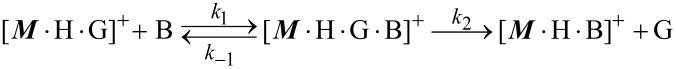


This profile indicates that, once formed, the [***M****∙*H∙G∙B]^+^ three-body complex can either back dissociate to [***M****∙*H∙G]^+^ and B (*k*_−1_), or “kick out” G in order to leave the protonated resorcin[4]arene coordinating to B (*k*_2_). The 10^11^*k*_1_/*k*_−1_ and *k*_−1_/*k*_2_ ratios are reported in Table 3.

**Table 3:** Rate constant ratios (Equation 6).

Complexes	10^11^*k*_1_/*k*_−1_ (cm^3^ molecule^−1^)		*k*_−1_/*k*_2_	

	B = **B^R^**	B = **B^S^**	B = **B^R^**	B = **B^S^**

[**V_R_***∙*H*∙*CT]^+^	2.4	0.5	4.6	21.5
[**V_S_***∙*H*∙*CT]^+^	1.1	0.9	7.6	12.7
[**V_R_***∙*H*∙*GC]^+^	18.4	23.4	6.2	5.3
[**V_S_***∙*H*∙*GC]^+^	4.3	7.6	22.2	4.1
[**V_R_***∙*H*∙*dC]^+^	<2 × 10^−3^	<2 × 10^−3^	>5 × 10^3^	>5 × 10^3^
[**V_S_***∙*H*∙*dC]^+^	0.5	0.5	25.1	24.4
[**V_R_***∙*H*∙*Cy]^+^	1.8	2.4	43.1	70.1
[**V_S_***∙*H*∙*Cy]^+^	6.4	9.8	1.3	2.1

The basicity of the nucleosides decreases in the order: dC < CT = Cy < GC, and in the same order the 10^11^*k*_1_/*k*_−1_ ratio tends to increase, most probably because the partial positive charge on the host pendants becomes larger, thus making the uptake of the third body B more efficient. The *k*_−1_/*k*_2_ ratio is invariably above unity, thus indicating that the release of B in general prevails on its uptake. Nevertheless, this ratio strongly depends on the electron demand of the 2’-substituent and on its orientation (Cy versus CT).

The more relevant result was observed for the complexes with G = dC as guest [55]. Indeed, when the host was in the *R-*configuration no reaction products were detected even after 300 s reaction time ([B] = 7.4 × 10^9^ molecule cm^−3^), whereas, under the same experimental conditions, the reaction carried out on the [**V_S_***∙*H∙dC]^+^ complex occurred and proceeded to over 20%. These findings indicate that the pre-equilibrium step involving [**V_R_***∙*H∙dC]^+^ (Equation 6) is ca. 200 times more shifted towards the reactants than that involving [**V_S_***∙*H∙dC]^+^ (dynamic range of the FT-ICR: ca. 10^3^:1). In other words the [**V_R_***∙*H∙dC]^+^ complex does not uptake B, whereas its diastereoisomers are able to efficiently capture B and to proceed to the nucleoside displacement.

**Figure 9:**
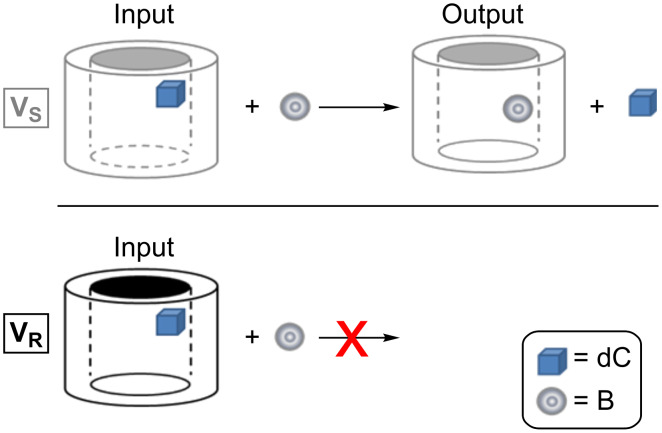
Example of molecular logic gate.

This very sensitive system strongly resembles the electronic concept of a logic gate. Indeed, depending on the relative configuration of the supramolecular device and of the neutral gas B, the transported nucleoside can or cannot be released. This effect is outstanding for the [**V_R/S_***∙*H∙dC]^+^ aggregates, which, if “stimulated” by a reactant characterized by the correct configuration, can selectively release one enantiomer of a chiral guest and keep the other enantiomer bound (Figure 9).

### Cyclochiral resorcin[4]arenes as chiral selectors

**Figure 10:**
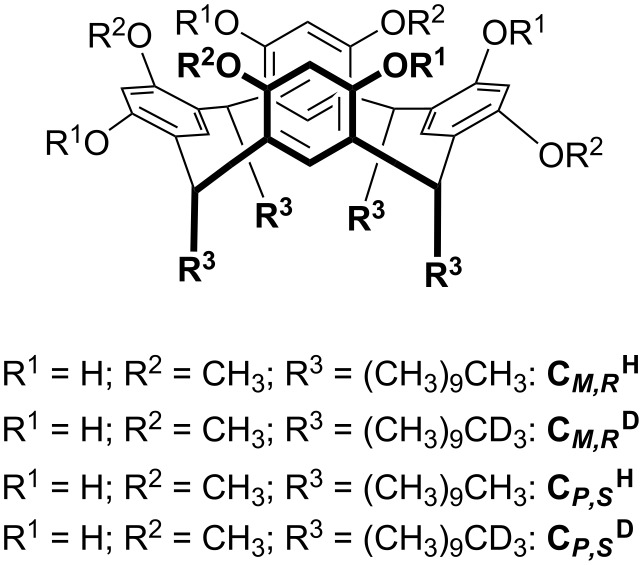
Cyclochiral resorcin[4]arenes.

A cyclochiral resorcin[4]arene is characterized by four identical subunits noncovalently coordinated by hydrogen bonds [56], three-dimensionally arranged either clockwise or counterclockwise. The authors investigated the effect of the mentioned source of asymmetry by generating, in a nanoESI-FT-ICR mass spectrometer, proton-bound complexes between resorcinarenes **C** (Figure 10) [57] and several polyfunctionalized biomolecules (G, Table 4), and then by measuring their gas-phase reactivity towards some primary amines (B: CH_3_CHΩNH_2_ with Ω = H, CH_3_, C_2_H_5_) [58].

**Table 4:** Overview of investigated amino acids, amino alcohols and amino acid esters.

Free AA^a^		Amino alcohols	

L-Phenylalanine	A_1_	L-Tyrosinol	N_1_
3,4-Dihydroxy-L-phenylalanine	A_2_	(1*R*,2*R*)-2-Amino-1-phenyl-1,3-propandiol	N_2_
L-Tryptophan	A_3_	L-Epinephrine	N_3_
5-Hydroxy-L-tryptophan	A_4_	L-Norepinephrine	N_4_
L-Tyrosine	A_5_		

AA ester			

L-Phenylalanine ethyl ester	E_1_		
L-Tyrosine methyl ester	E_2_		

^a^amino acid.

The presence of four decamethylene lateral chains in the structure of **C** favored the arrangement of guests on the upper aromatic cavity of **C**, and the remote position of labeled or unlabeled methyl groups enables the kinetic measurements of the corresponding quasi-diastereomeric complexes under the same experimental conditions (Equation 8 and Equation 9; B: CH_3_CHΩNH_2_ with Ω = H, CH_3_, C_2_H_5_) [59–60].

[8]
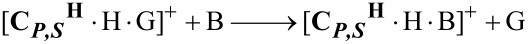


[9]
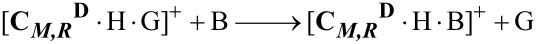


The selected [**C***∙*H∙G]^+^ complexes exist as single kinetically distinguishable structures. The heterochiral complexes mostly react faster than their homochiral homologues, with the exception of the [**C***∙*H∙G]^+^ complexes with G = A_4_, N_2_, N_4_ (Table 5) [58].

**Table 5:** *k*_homo_/*k*_hetero_ measured for reactions of [**C^H^***∙*H∙G]^+^/[**C^D^***∙*H∙G]^+^ complexes with **B**.

	Ω = H	Ω = CH_3_	Ω = C_2_H_5_	Ω = C_2_H_5_ (*S*)-

P.A.	210	212.5	214.1	214.1

**G**	*k*_homo_/*k*_hetero_ (ρ)			

A_1_	0.99 ± 0.07	—	1.10 ± 0.04	1.10 ± 0.08
A_2_	0.39 ± 0.01	0.70 ± 0.05	0.93 ± 0.06	0.94 ± 0.04
A_3_	0.56 ± 0.02	—	0.95 ± 0.06	1.08 ± 0.07
A_4_	—	2.03 ± 0.11	1.31 ± 0.08	1.44 ± 0.09
A_5_	—	—	0.95 ± 0.05	0.99 ± 0.06

E_1_	0.53 ± 0.03	0.74 ± 0.03	0.77 ± 0.03	0.82 ± 0.08
E_2_	—	0.80 ± 0.04	0.82 ± 0.04	0.84 ± 0.05

N_1_	0.59 ± 0.03	0.47 ± 0.02	0.54 ± 0.03	0.76 ± 0.03
N_2_	—	3.89 ± 0.29	3.94 ± 0.23	2.65 ± 0.38
N_3_	—	0.89 ± 0.04	1.02 ± 0.04	0.78 ± 0.03
N_4_	—	1.51 ± 0.09	1.57 ± 0.09	1.32 ± 0.08

As previously mentioned, the origin of the enantioselectivities showed in Table 5 can be due to a thermodynamic and/or kinetic control of the reaction coordinates. It has been found that when the proton affinity of the neutral amine increases, and the reaction becomes enthalpically favored, the measured enantioselectivity tends to decrease. This finding indicates that the main reaction control factor is the kinetic one, because the system is less enantioselective if the involved transition structures become more similar to the starting diastereomeric reactant complexes, by following the proton affinity order of B. This interpretation was supported by independent MS tandem measurements carried out on the [**C*_M_*_,_*_R_*^a^***∙***C*_P_*_,_*_S_*^b^**∙H∙G]^+^ (G = N_1_; N_2_; a,b = H,D or D,H) ternary adducts yielding [**C*_M_*_,_*_R_*^a^***∙*H∙G]^+^ and [**C*_P_*_,_*_S_*^b^**∙H∙G]^+^ as the fragmentation products. The distribution of the quasi-enantiomer fragments points to the same relative stability for the starting diastereomeric [**C**∙H∙N_1_]^+^ complexes and an appreciable energy difference between the diastereoisomers of [**C**∙H∙N_2_]^+^ ([**C**∙H∙N_2_]^+^_hetero_ > [**C**∙H∙N_2_]^+^_homo_). The combination of the dependence of ρ on the proton affinity of the amine and the independent CID data clearly indicates that the kinetics of the [**C**∙H∙N_1_]^+^ adducts is mostly controlled by the cyclochirality determining the differential energies of the involved transition structures. In contrast, the significant enantioselectivity exhibited by the diastereomeric [**C**∙H∙N_2_]^+^ complexes is due to the synergy between their different stabilities and the effect of the asymmetric architecture of the cyclochiral resorcin[4]arene.

The presented evidence represents the first example of a kinetic enantiorecognition mainly due to an exclusive structural factor: the cyclochirality of the receptor’s cavity.

## Conclusion

The high enantioselectivity found in biochemical systems is essentially due to several intimate noncovalent interactions. In living systems a covalent bond between a neurotransmitter and its macromolecular target cannot be imagined, because it would produce an irreversible inactivation of the receptor primary functions, which is incompatible with the existence of living matter itself. In fact, the pharmacological mechanism, so called suicide, of many drugs (e.g., anticancer molecules) consists in the formation of a covalent, and thus irreversible, bond with their target. The main advantage of a gaseous environment is the exclusion of any counterion and/or solvent effect, while at the same time the chemicophysical properties of the isolated system can be subtly studied.

Finally, the application of the displacement-reaction methodology provides a variety of information on the dynamic behavior of a supramolecular device: (1) The decay curve of a selected precursor indicates whether one or several kinetically distinguishable structures exist; (2) The effect of the neutral configuration suggests the actual location of the substituted guest (external or internal); (3) the effect of the neutral proton affinity on the measured enantioselectivity indicates the prevalence of kinetic or thermodynamic reaction control. Further energetic details can be gained by an independent mass-spectrometric approach (Cook’s method on a three-body complex), and by several computational supports (molecular dynamics and ab initio optimization). The dynamic point of view is fundamental for supramolecular ionic aggregates, because the synergy of several noncovalent interactions confers a pronounced stability even to very flexible aggregates, and the lifetime of the same interactions determines the reaction pathway.

The reviewed papers point to the crucial role of the nature and sequence of the resorcin[4]arene pendants, even in the case of gas-phase biorecognition, analogous to the enzymatic behavior. Furthermore, the high selectivity in the reaction of [***M***∙H∙drug]^+^ towards an organic base could efficiently reproduce the driving forces for the intimate contact between the same drug and its biotarget.
